# Structural and Functional Analysis of Porcine CR1-like Proteins in C4b-Mediated Immune Responses

**DOI:** 10.3390/vetsci13010033

**Published:** 2025-12-30

**Authors:** Wei Yin, Nan Wang, Jingze Li, Haoxiang Yao, Qiongyu Li, Hongquan Li, Kuohai Fan, Jia Zhong, Zhenbiao Zhang, Na Sun, Panpan Sun, Huizhen Yang, Jianzhong Wang, Yaogui Sun

**Affiliations:** Shanxi Key Laboratory for Modernization of TCVM, College of Veterinary Medicine, Shanxi Agricultural University, Jinzhong 030801, China; 202430936@stu.sxau.edu.cn (N.W.); 202531036@stu.sxau.edu.cn (J.L.); 202531098@stu.sxau.edu.cn (H.Y.); 20233832@stu.sxau.edu.cn (Q.L.); livets@163.com (H.L.); fkhyxj@163.com (K.F.); zhongjia3294@163.com (J.Z.); zbzhangvet@sxau.edu.cn (Z.Z.); snzh060511@126.com (N.S.); sunpp0505@163.com (P.S.); hzyang2020@163.com (H.Y.); wjz2020@foxmail.com (J.W.); dkyypb@163.com (Y.S.)

**Keywords:** PRRSV, immune adhesion, CR1-like

## Abstract

The complement system is crucial for immune defense, linking innate and adaptive immunity. In the classical and lectin pathways, C4 is split into C4b, triggering opsonization, lysis, and the removal of pathogens and damaged cells. Dysregulated activation of C4 and other components of the classical pathway can lead to tissue damage and heightened inflammation, whereas appropriate regulation of C4b activity serves to mitigate excessive inflammation and prevent injury. ELISA analysis demonstrated C4 activation and cleavage during the co-incubation of PRRSV with fresh porcine serum. Immunoelectron microscopy revealed that porcine red blood cells could immunologically adhere to PRRSV, and C4b was involved in this adhesion process. BLAST (NCBI BLAST+ 2.14.1) analysis revealed that porcine CR1-like CCPs 1-3, CR1-like CCPs 12-14, and CR1-like CCPs 19-21 share high similarity with the CCP 1-3 region of human CR1, which mediates C4b binding. Yeast two-hybrid assays confirmed that all three CR1-like fragments bind C4b. To elucidate the interaction mechanism, homology models of C4b and CR1-like fragments were constructed, followed by molecular docking and dynamics simulations, identifying 18 key amino acids in porcine CR1-like involved in C4b binding. Surface plasmon resonance further validated the binding affinity of CR1-like CCPs 1-3, its mutant 118I, and C4b. These results enhance our understanding of complement regulation and provide a foundation for developing therapeutic strategies targeting complement-related diseases.

## Simple Summary

The complement system is crucial for innate immunity, but its role against Porcine Reproductive and Respiratory Syndrome Virus (PRRSV), a significant swine pathogen, is not well understood. This study investigated the interaction between porcine CR1-like protein and C4b in PRRSV immunity. ELISA showed PRRSV triggers C4 cleavage, and immunoelectron microscopy indicated C4b facilitates porcine red blood cell adhesion to PRRSV. Yeast two-hybrid assays identified three CR1-like fragments binding C4b, structural simulations pinpointed 18 key amino acids, and SPR confirmed that the 118I mutation reduced affinity between CCPs 1-3 and C4b. These findings reveal that porcine CR1-like protein aids immune adhesion to PRRSV through C4b binding, clarify the structural basis, improve understanding of porcine complement defense, and lay the groundwork for PRRSV treatments.

## 1. Introduction

The complement system constitutes a fundamental element of innate immunity, playing a crucial role in combating microbial infections, eliminating foreign pathogens, and preserving tissue homeostasis. Activation of the complement system induces an upregulation of cytokines, chemokines, and other molecules integral to the innate immune response [1,2]. The complement component C4, with a molecular weight of 204 kDa, is a crucial part of the complement system and is essential in the classical and lectin pathway cascades. It is a three-chain glycoprotein linked by disulfide bonds, consisting of α chains (95 kDa), β chains (75 kDa), and γ chains (30 kDa) [3,4]. C4b is a product of C4 cleavage and is produced by activation of the C1q, C1r, and C1s complex (classical pathway) and mannan-binding lectin (MBL) [5,6]. Through its reactive thioester, C4b binds to the target surface and can be transformed into the intermediate iC4b form, undergoing proteolysis by serine protease factor I and cofactor CD46 [7,8]. Further cleavage of iC4b yields thioester-linked C4d (45 kDa) and soluble C4c (146 kDa), which can act as biomarkers for complement activation in the classical and lectin pathways. The risk of infections and autoimmune diseases can rise with a complete or partial lack of complement C4. Many studies have indicated that complement C4 is essential for protection against microbial infections [9]. Increased vulnerability to infection is associated with a full or partial deficiency of complement C4 [10]. Additionally, an insufficiency of complement C4 might cause several autoimmune disorders [11]. Decreased C4 protein concentration and decreased serum complement activity are common in systemic lupus erythematosus (SLE) and other active infectious diseases. The change in C4 content in plasma can also be used as an indicator to monitor various diseases [12,13,14,15]. On the surface of Group B Streptococcus (GBS), the complement-interfering protein (CIP) enhances cell adhesion and penetration and disrupts the host’s innate and adaptive immune systems. Findings suggest that CIP can associate with human C4b ligands, impacting the classical and lectin complement pathways [16]. Bordetella pertussis (Gram-negative bacteria) can evade attack by the complement system by releasing the virulence protein Vag8. Recombinant Vag8, when secreted endogenously, can hinder complement deposition on bacterial surfaces at the C4b level [17]. Recent studies have shown that flavivirus non-structural (NS1) proteins of dengue virus (DENV), West Nile virus (WNV), and yellow fever virus (YFV) are capable of attaching to C4, promoting its cleavage, reducing the deposition of C4b, and decreasing the activity of the C3 converting enzyme (C4bC2a), and thus confer a viral immune escape function. A recent study has uncovered a new antiviral mechanism that relies on C4 but not on late-acting complement components. C4 impedes human adenovirus infection by depositing lysed C4b onto the capsid, preventing its decomposition, release from endosomes, and infiltration into the cytoplasm [18]. Increased C4b may exacerbate tissue damage through inflammatory responses, suggesting that C4b could serve as a prognostic biomarker for type 1 diabetes. Severe bacterial infections can cause tissue destruction, cavity formation, and activation of the complement system.

Expressed on red blood cells and immune cells, CR1 is a type I membrane glycoprotein that centrally regulates the classical pathway, lectin pathway, and replacement pathway of the complement system [19,20,21,22,23]. The structure includes 30 closely related domains known as short consensus repeats (SCRs), complement control protein (CCP), or Sushi domain, in addition to a transmembrane domain and a cytoplasmic tail consisting of 43 amino acids [24]. SCR domains 1–28 are organized into four larger units, each consisting of 7 domains, known as long homologous repeat sequences (LHR), labeled LHR-A to LHR-D [25]. In LHR-A, LHR-B, and LHR-C, the initial three CCPs of each LHR facilitate interactions with either C3b or C4b. SCRs 1-3 mainly bind to C4b and have a weak affinity for C3b. CCPs 8-10 and CCPs 15-17 mainly bind to C3b but also have some affinity for C4b. In the genetic sequence of human CR1, CCPs 8-10 and CCPs 15-17 are more than 99% similar, which means that their structure and function are almost identical [26]. This study analyzed changes in C4 levels in porcine fresh serum at different time points within a co-incubation system using ELISA technology. Immune electron microscopy was employed to observe whether C4b participates in the immune adhesion of red blood cells to Porcine Reproductive and Respiratory Syndrome Virus (PRRSV). The yeast two-hybrid technique was utilized to detect whether CR1-like binds to C4b. By combining homology modeling, molecular docking, and dynamics simulations, an interaction model between porcine CR1-like and C4b was constructed and optimized, further establishing the kinetic parameters of their interaction and elucidating their structural characteristics. Finally, protein expression and surface plasmon resonance (SPR) technology were used to validate the biological activity of the binding region between CR1-like and C4b. This study aims to provide a theoretical foundation for further revealing the mechanism of porcine red blood cell immune adhesion and its relationship with diseases, as well as to expand the in-depth understanding of the complement system’s functions.

## 2. Materials and Methods

### 2.1. Animals and Strains

Three healthy female 40-day-old Landrace piglets, each weighing approximately 20 kg, were obtained from Taigu County Guannong Agriculture and Animal Husbandry Technology Co, Ltd., Taigu, China. Our lab cryopreserved the PRRSV isolate JS-1 with a TCID_50_ of 10^−5.55^/mL.

### 2.2. Detection of Changes in Serum C4 Activity

Three experimental groups were set: group I (fresh pig serum + PBS), group II (fresh pig serum + PRRSV), and group III (inactivated pig serum + PRRSV). All groups were incubated at 37 °C; supernatants were collected via centrifugation at 0, 0.5, 1, 2, and 3 h post-incubation. C4 activity in supernatants was measured using a pig C4 ELISA kit (Shanghai Jianglai Biotechnology Co., Ltd., Shanghai, China), and optical density (OD) values were recorded. Statistical analysis was performed via one-way ANOVA using GraphPad PrismTM 8 (GraphPad Software, San Diego, CA, USA).

### 2.3. C4b in EM Detection of RBC-PRRSV Adhesion

Group A served as the cell control group, where porcine red blood cells were co-incubated with PBS. Group B was the virus blank group: 5 mL of PRRSV suspension was mixed with 5 mL of 4% paraformaldehyde (Wuhan Servicebio Technology Co., Ltd., Wuhan, China) and fixed by suspension at 4 °C. Group C was the virus control group, where PRRSV was first co-incubated with PBS and then with porcine red blood cells. Group D was the sensitized virus group, where PRRSV was first co-incubated with fresh porcine serum and then with porcine red blood cells. Group E was the inactivated serum group, where PRRSV was first co-incubated with inactivated porcine serum and then with porcine red blood cells. Group F was the antibody blockade group: 5 mL of porcine red blood cells were incubated with CR1-like antibody (laboratory-made, Patent No.: ZL201410308534.0) at 37 °C for 2 h, while 5 mL of PRRSV suspension was incubated with 250 μL of fresh porcine serum at 37 °C for 1 h and then mixed with 5 mL of porcine red blood cells and incubated at 37 °C for 30 min. The samples were sequentially centrifuged, fixed with glutaraldehyde (0.5%, 4 °C for 3 min; 3%, 4 °C for 10 h, Sigma-Aldrich, St. Louis, MO, USA), rinsed with PBS, dehydrated with an acetone gradient (50–100%,Sigma-Aldrich, St. Louis, MO, USA), and embedded in resin. Ultrathin sections of 80 nm were prepared, etched with hydrogen peroxide (Sigma-Aldrich, St. Louis, MO, USA) for 30 min, and then sequentially washed with PBS, blocked with BSA(Beijing Solarbio Science & Technology Co., Ltd., Beijing, China), incubated with C4b monoclonal antibody (Abcam plc, Cambridge, UK) at 4 °C for 20 h, and incubated with colloidal gold-labeled secondary antibody (Beijing Zhongshan Jinqiao Biotechnology Co., Ltd., Beijing, China) at room temperature for 2 h. Finally, the samples were double-stained with uranyl (Sigma-Aldrich Production GmbH, Buchs, Switzerland) acetate and lead citrate(Sigma-Aldrich Production GmbH, Buchs, Switzerland), dried, and observed under a transmission electron microscope (80 kV).

### 2.4. Pig CR1-like-C4b Domain Prediction

Amino acid sequences were retrieved from databases: human CR1 (UniProt: P17927), pig CR1-like (GenBank: NC_010451.4), human C4b (UniProt: P0C0L4), and pig C4b (GenBank: NC_010449.5). Domain annotation: Human CR1 and pig CR1-like sequences were uploaded to SMART (Letunic et al., 2021, mode: NORMAL) [27] to identify CCP domains; human CR1 C4b-binding domains (CCPs 1-3) were used for BLAST (National Center for Biotechnology Information, Bethesda, MD, USA) homology analysis against pig CR1-like to screen homologous pig CR1-like fragments. Signal peptide and transmembrane prediction: target sequences (pig CR1-like fragments and C4b) were analyzed via SignalP 6.0 [28] (species: eukaryotes) and DeepTMHMM-1.0 [29] to retain extracellular regions without signal peptides (to reduce computational complexity).

### 2.5. Construction of Recombinant Plasmid

Recombinant plasmids (pGBKT7-CR1-like CCPs 1-3/12-14/19-21; pGADT7-C4bα/β/γ) were synthesized by Sangon Biotech using pGBKT7/pGADT7 vectors (TaKaRa (Shanghai, China)). Plasmids were verified via double digestion and Sanger sequencing.

### 2.6. Self-Activation Detection and Toxicity Test

Self-activation and toxicity of recombinant plasmids were tested using the Yeastmaker™ Yeast Conversion System 2 kit (TaKaRa): plasmids included pGBKT7-derived (pGBKT7-CR1-like CCPs 1-3/12-14/19-21, empty pGBKT7) and pGADT7-derived (pGADT7-C4bα/β/γ, empty pGADT7), which were transformed into Y2HGold yeast (Angyu Biotechnology Co., Ltd., Shanghai, China) and cultured on selective plates for 2–4 days (pGBKT7: SD/-Trp, SD/-Trp/X-α-Gal, SD/-Trp/X-α-Gal/AbA; pGADT7: SD/-Leu, SD/-Leu/X-α-Gal, SD/-Leu/X-α-Gal/AbA); non-self-activation was verified by white colonies on SD/-Trp/SD/-Leu and SD/-Trp/X-α-Gal/SD/-Leu/X-α-Gal plus no colonies on SD/-Trp/X-α-Gal/AbA/SD/-Leu/X-α-Gal/AbA, non-toxicity by recombinant plasmid colonies matching empty vector colonies in size, and only non-self-activating, non-toxic plasmids were used for subsequent experiments.

### 2.7. Identification of CR1-like Active Fragment Combined with C4b Yeast Two-Hybrid

pGBKT7-CR1-like and pGADT7-C4b were transformed into the same yeast strain Y2HGold. The successfully transformed single colony was selected and cultured in 5 mL of YPDA at 30 °C and 250 rmp for 24 h. The bacteria solution was diluted and coated on SD/-Leu, SD/-Leu/-Trp (DDO), SD/-Leu/-Trp/X-α-Gal (DDO/X), and SD/-Leu/-Trp/X-α-gal/Aba (DDO/X/A) plates for 2~4 days at 30 °C to observe the growth of the colony and the growth color of the colony. Y2HGold transformed using pGBKT7-53 vector was mated with Y187 cells containing pGADT7-T and used as a positive control. Y2HGold transformed using pGBKT7-Lam vector was mated with Y187 cells containing pGADT7-T and used as a negative control.

### 2.8. Homology Modeling

All templates for protein crystal structures in homology modeling came from the PDB database (RCSB Protein Data Bank, https://www.rcsb.org/), and AlphaFold (DeepMind) [30] was employed to forecast the 3D structure of C4b and CR1-like CCPs 1-3. The model quality was assessed using the SAVES v6.0 (RCSB Protein Data Bank) web server and the PROCHECK (EMBL-European Bioinformatics Institute) [31] module of the ProSA (University of Vienna) [32] online inspection tool. CR1-like CCPs 12-14 and CR1-like CCPs 19-21 used the optimized model of Hou [33].

### 2.9. Molecular Docking and Kinetic Simulation

The Gramm-X v2.0 (University of Groningen) [34] online server was used for free docking of CR1-like CCPs 1-3, CR1-like CCPs 12-14, and CR1-like CCPs 19-21 with C4b, respectively. The optimal docking results were selected for molecular dynamics simulation. Desmond/Maestro (2022.1, D. E. Shaw Research; Schrödinger, Inc. (New York City, NY, USA)) was selected as the kinetic simulation software, TIP3P was added to the complex system using the TIP3P water model, and 0.15 M of sodium chloride solution (Sigma-Aldrich, St. Louis, MO, USA) was added to the equilibrium system. After minimizing and relaxing the system, 100 ns molecular dynamics simulations were executed in an isothermal isobaric ensemble at 300 K and 1 bar. Every 100 ps, the coordinates of the track were recorded, and Desmond’s simulated interaction diagram was employed for molecular dynamics analysis to review trajectory files and output model files with the least energy. Using PRODIGY v2.0 (University of Warsaw) [35], the affinity of proteins that interacted was calculated.

### 2.10. Prediction of Key Amino Acids in CR1-like-C4b Interactions

To analyze the interaction interface between CR1-like and C4b, LIGPLOT v4.5.3 (EMBL-European Bioinformatics Institute) was utilized, identifying the residues and types of interactions. DIMPLOT v4.5.3 (EMBL-European Bioinformatics Institute)was selected as the analysis mode for analyzing protein–protein interactions. The complex was further scanned with DrugScorePPI v1.0 (University of Düsseldorf) [36], mCSM-PPI2 v1.1 (King’s College London) [37], Robetta v3.0(University of Washington Baker Laboratory) [38], and BAlaS v1.2 (University of Edinburgh; University of Bristol) [39,40] to mimic alanine mutation. These tools predicted key residues of the complex by simulating alanine mutation scanning and calculated the change value of binding free energy before and after the mutation of residues. The key amino acid binding site was determined when the binding free energy change was more than 1 kcal mol^−1^.

### 2.11. C4b Is Expressed in the Large Intestine Expression System

The pET28a vector was employed to express C4b in the *Escherichia coli* expression system, incorporating 6His and Flag-ppase tags at the N-terminus while leaving the C-terminus untagged, thereby constructing the recombinant plasmid. The plasmid was transferred into *Escherichia coli* for expression, purified by preloaded Ni-NTA column, eluted with different concentrations of imidazole solution, and the fractions were collected and concentrated. Subsequently, further purification was performed using a strong cation exchange column (SP-HP), the fractions were equilibrated with 30 mM MES buffer (pH 6.5), 1 mM TCEP, and 5% glycerol, and the flow-through fraction was collected by continued washing with the same buffer after loading. Finally, fractions were collected and analyzed by SDS-PAGE after 0% to 100% gradient elution with 30 mM MES buffer (pH 6.5), 1 mM TCEP, 5% glycerol, and 1.0 M NaCl.

### 2.12. CR1-like Fragments and Their Mutants Are Expressed in Insect Cells

Recombinant plasmids were constructed utilizing pFastBac as a vector, incorporating 6His and sumo tags at the N terminus and flag tags at the C terminus. Recombinant baculoviruses were subsequently generated through the Bac-to-Bac system and transfected into SF9 insect cells for expression. Culture supernatants containing CR1-like fragments or their mutants were collected by centrifugation 72 h after infection. Purification was performed using a preloaded Ni-NTA column, and the fractions were collected and concentrated by elution through various concentrations of imidazole solutions. Further purification was carried out using a molecular sieve SEC column P200 10/500. The column was first equilibrated with 50 mM Tris buffer (pH 8.0), 150 mM NaCl, 1 mM TCEP, and 10% glycerol and eluted with the same buffer after sample loading. Fractions were collected according to the time or volume of the elution peak and analyzed by SDS-PAGE to identify fractions containing recombinant proteins.

### 2.13. Surface Plasmon Resonance (SPR) Detection

Single-cycle interaction analysis was performed on a Biacore 8K instrument (Cytiva (Marlborough, MA, USA)) with C4bα and C4bβ covalently immobilized on a CM5 sensor chip with an amount of approximately 1500 RU. Experiments were performed at 25 °C with a binding time of 60 s, a dissociation time of 300 s, and a flow rate of 30 μL/min. The running buffer used was 10 mM HEPES (pH 7.4), 150 mM NaCl, and 0.05% Tween-20. The size of CR1-like CCPs 1-3, CR1-like CCPs 12-14, and their mutants were 1.56 × 10^−2^ μM, 3.12 × 10^−2^ μM, 6.24 × 10^−2^ μM, 0.125 μM, 0.25 μM, 0.5 μM, 1 μM, 2 μM, and 4 μm, respectively. The concentration of μM was used as the mobile phase to detect the binding of C4bα and C4bβ to CR1-like CCPs 1-3, CR1-like CCPs 12-14, and their mutants, respectively. The apparent equilibrium dissociation constant (KD) was calculated as the ratio of dissociation and binding rate constants (kd/ka), with smaller KD values indicating stronger affinity.

## 3. Results

### 3.1. PRRSV Activates C4 and C4b Mediates RBC-PRRSV Immune Adhesion

**Figure 1:**
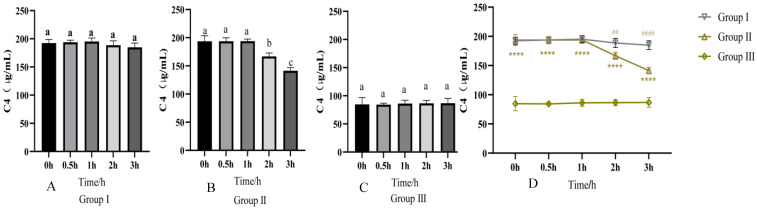
Results of changes in serum C4 activity. (**A**) Represents the results of C4 activity in the group incubated with fresh serum and PBS. (**B**) Represents the results of C4 activity in the group incubated with fresh serum and PRRSV. (**C**) Represents the results of C4 activity in the group incubated with inactivated serum and PRRSV. (**D**) Represents the statistical results of the change in C4 activity in different groups. Group I was the group with fresh serum and PBS; group II was the group with fresh serum and PRRSV; and group III was the group with inactivated serum and PRRSV. Different letters indicate differences between groups (*p* < 0.05). **** indicates that there is a significant difference between group II and group III (*p* < 0.001), ## indicates that there is a significant difference between group II and group I (*p* < 0.05), and #### indicates that there is a significant difference between group II and group I (*p* < 0.001).

Regarding the activation of complement C4 by PRRSV, the results showed that group I (fresh serum + PBS) had stable C4 activity in the first hour, with a non-significant slight decrease at 1–3 h (*p* > 0.05); group II (fresh serum + PRRSV) showed stable C4 activity initially but a gradual decline at 1–3 h; group III (inactivated serum + PRRSV) had nearly unchanged C4 activity. Statistics confirmed significant inter-group differences (*p* < 0.05), with group II having lower C4 activity than group I (*p* < 0.05, *p* < 0.001) and group III (*p* < 0.001), indicating PRRSV activates C4, leading to its cleavage and reduced activity at 1–3 h (Figure 1).

**Figure 2:**
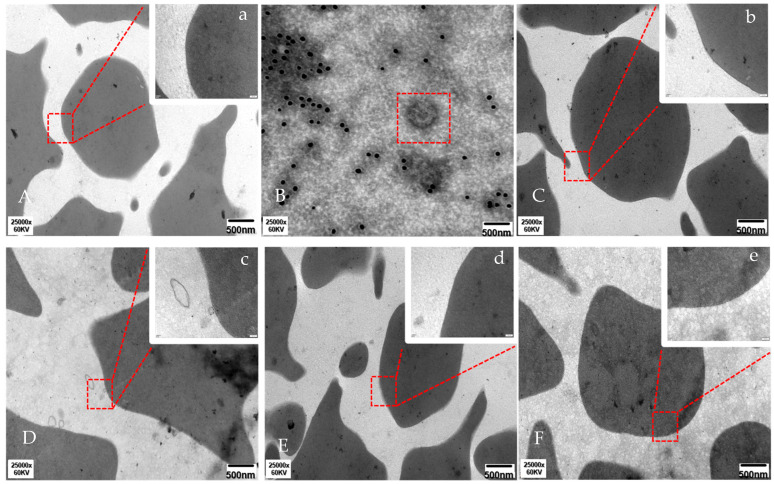
Results of colloidal gold particle electron microscopy detection for C4b in different groups. (**A**) Represents the porcine red blood cell control group. (**B**) Represents the virus blank group. (**C**) Represents the virus control group. (**D**) Represents the fresh serum incubation group. (**E**) Represents the inactivated serum incubation group. (**F**) Represents the antibody blocking group. In Figure (**A**–**F**), bar = 500 nm and magnification 25,000×. In Figure (**a**–**e**), bar = 100 nm and magnification 120,000×. The red arrow indicates colloidal gold particles between PRRSV and pig erythrocyte.

The findings in Figure 2A show that porcine red blood cells (RBCs) appeared normal with elliptical or varied shapes under transmission electron microscopy. Figure 2B highlights PRRSV in a red box. Figure 2C shows similar RBC morphology to Figure 2A, without immune adherence. In Figure 2D, immune adherence was observed, with colloidal gold particles indicating binding between the RBC membrane and PRRSV, involving C4b. Figure 2E,F show no RBC immune adherence. These results indicate that C4b plays a role in the immune adherence of porcine RBCs to PRRSV.

### 3.2. Bioinformatics Prediction of CR1-like-C4b Interaction Domains

**Figure 3:**
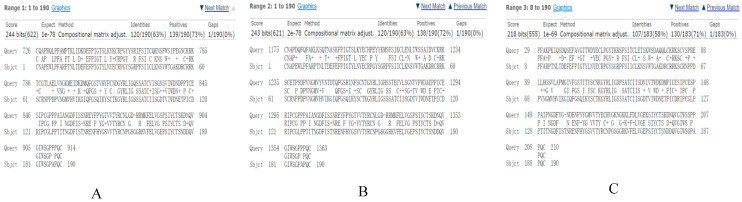
Sequence alignment of human CR1 CCPs 1-3 and pig CR1-like. Query is the porcine CR1-like protein sequence, and Sbjct is the human CR1 CCPs 1-3 sequence. (**A**) Shows the segment with the highest similarity between pig CR1-like and human CR1 CCPs 1-3. (**B**) Shows the segment with the second highest similarity of pig CR1-like protein sequence to human CR1 CCPs 1-3. (**C**) Shows the segment with the third highest similarity of pig CR1-like protein sequence to human CR1 CCPs 1-3.

BLAST alignment of the C4b-binding domain (CCPs 1-3) of human CR1 with porcine CR1-like identified three homologous fragments: CR1-like CCPs 1-3 (58% identity), CCPs 12-14 (63% identity), and CCPs 19-21 (63% identity). These fragments were selected as potential C4b-binding targets for subsequent experimental validation (Figure 3). Additionally, SignalP 6.0 and DeepTMHMM-1.0 confirmed that C4b and all three CR1-like fragments are extracellular regions without signal peptides, ensuring their spatial accessibility for in vivo interaction. Porcine CR1-like was also found to contain 31 CCP domains, showing structural conservation with human CR1 (37 CCP domains), which supports the hypothesis that porcine CR1-like retains C4b-binding activity.

### 3.3. Yeast Two-Hybrid Validation of CR1-like-C4b Interaction

Recombinant plasmids for yeast two-hybrid assays were first verified by double digestion and Sanger sequencing, with inserted fragments matching the expected sizes (557 bp for CR1-like CCPs 1-3/12-14, 725 bp for CCPs 19-21, and 1968 bp/2040 bp/885 bp for C4b α/β/γ chains). Toxicity assays confirmed no inhibitory effect of these plasmids on Y2HGold yeast growth, and self-activation assays ruled out non-specific reporter gene activation by either pGBKT7 or pGADT7 recombinant plasmids, ensuring the reliability of the assay system.

**Figure 4:**
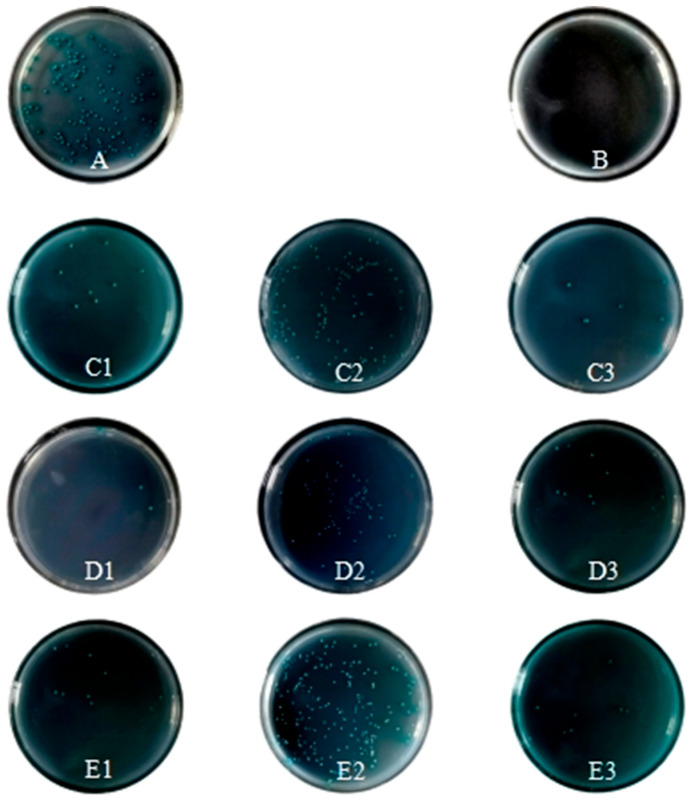
Presents yeast two-hybrid interactions between CR1-like CCP fragments and C4b subtypes on DDO/X/A medium. (**A**) Shows the positive control (pGBKT7-53 + pGADT7-T), and (**B**) shows the negative control (pGBKT7-Lam + pGADT7-T). (**C1**–**C3**) represent interactions involving pGBKT7-CR1-like CCPs 1-3 with pGADT7-C4b α/β/γ; (**D1**–**D3**) involve CCPs 12-14; and (**E1**–**E3**) involve CCPs 19-21.

Specific binding between CR1-like fragments and C4b chains was confirmed on the stringent DDO/X/A medium (supplemented with AbA to eliminate non-specific binding). Blue colonies were observed in all experimental groups (CR1-like CCPs 1-3/12-14/19-21 paired with C4b α/β/γ chains), while the negative control (pGBKT7-Lam + pGADT7-T) showed no colony growth. Colonies in the CR1-like CCPs 1-3 + C4b α and CR1-like CCPs 12-14 + C4b β groups exhibited more vigorous growth, indicating stronger binding affinity between these pairs (Figure 4).

### 3.4. Molecular Simulation of CR1-like-C4b Interaction and Key Amino Acid Prediction

**Figure 5:**
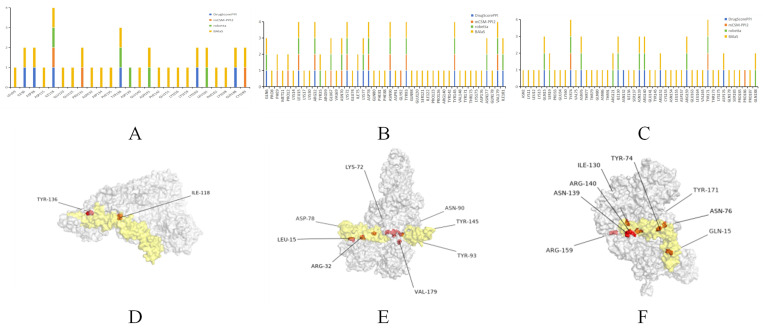
Prediction and display of hot spot residues in C4b-CR1-like CCPs 1-3, 12-14, and 19-21 complexes. (**A**–**C**) Show hot spot residues at the respective binding interfaces predicted by four tools. (**D**–**F**) Show the display of hot spot residues for each complex.

Key amino acids critical for CR1-like-C4b binding were predicted using four independent tools (DrugScorePPI, mCSM-PPI2, Robetta, and BAlaS). For CR1-like CCPs 1-3, Ile118 and Tyr136 were validated by at least three tools; for CCPs 12-14, eight residues (including Leu15 and Arg32) were validated by at least four tools; and for CCPs 19-21, eight residues (including Gln15 and Tyr74) were validated by at least three tools. The spatial locations of these key residues further confirmed their direct involvement in the binding interface (Figure 5). Three-dimensional models of porcine CR1-like CCPs 1-3 and C4b were constructed using AlphaFold2 and validated as reliable by ProSA (normal Z-scores) and PROCHECK (core region residues > 89%), and molecular dynamics simulations confirmed the structural stability of C4b-CR1-like complexes.

### 3.5. Recombinant Protein Expression and SPR Validation of CR1-like-C4b Binding Affinity

**Figure 6:**
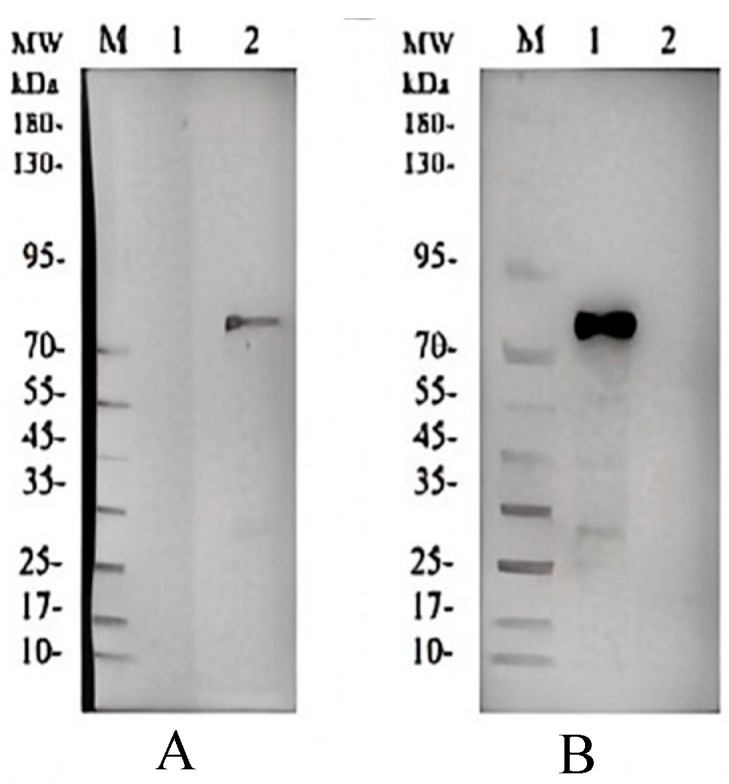
Western blot verification of C4bα and C4bβ proteins. (**A**) Western blot result of C4bα: M represents the protein marker, 1 is the negative control, and 2 is the C4bα protein fraction eluted with 400 mM imidazole. (**B**) Western blot result of C4bβ: M represents the protein marker, 1 is the C4bβ protein fraction eluted with 400 mM imidazole, and 2 is the negative control. Western Blot original pictures see Figures S1–S3.

Recombinant C4b α/β chains were expressed in *Escherichia coli* and purified via Ni-NTA and strong cation exchange chromatography (SP-HP). Western blot analysis of 400 mM imidazole eluates confirmed the presence of the Flag tag and expected molecular weights (70–95 kDa) for both C4b α and β chains, verifying high purity and suitability as binding substrates (Figure 6).

**Figure 7:**
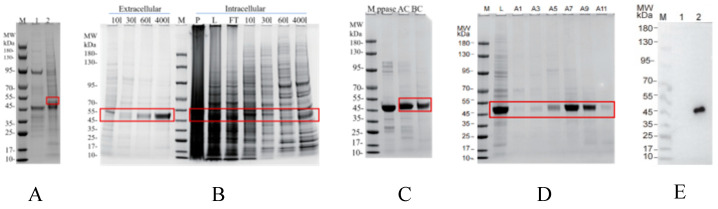
Purification of CR1-like CCPs 1-3. (**A**) Shows the expression analysis of CR1-like CCPs 1-3. In this panel, M represents the protein marker, 1 indicates the negative control, and 2 corresponds to CR1-like CCPs 1-3. The negative control (lane 1) refers to lysate of SF9 insect cells transfected with the empty pFastBac vector (without the CR1-like CCP 1-3 insert). This control excludes signals from the vector backbone, insect cell endogenous proteins, or antibody cross-reactivity unrelated to the target protein. (**B**) Presents the purification results of CR1-like CCPs 1-3 using a Ni column. In this panel, extracellular refers to the supernatant collected from insect cell culture and Intracellular represents the lysed insect cells. M denotes the protein marker, P indicates the pellet obtained after centrifugation of the lysed insect cells, L is the supernatant obtained post-centrifugation, and FT refers to the flow-through fraction. The labels 10l, 30l, 60l, and 400l correspond to eluates obtained with 10 mM, 30 mM, 60 mM, and 400 mM imidazole, respectively. (**C**) Illustrates the purification results of CR1-like CCPs 1-3 following PPASE protease cleavage. Here, BC represents the sample before cleavage, AC indicates the sample after cleavage, and PPASE denotes the protease. M is the protein marker. (**D**) Shows the purification results of CR1-like CCPs 1-3 using SEC. In this panel, M refers to the protein marker, L represents the supernatant, and A1–A11 are fractions collected based on elution time or volume. (**E**) Shows the Western blot results of the concentrated components; lane 1 represents the negative control and lane 2 represents CR1-like CCPs 1-3. Western Blot original pictures see Figures S1–S3.

Recombinant CR1-like CCPs 1-3 were expressed in insect cells using the Bac-to-Bac system and purified via Ni-NTA and size-exclusion chromatography (SEC). Western blot analysis of concentrated SEC fractions showed a specific Flag tag signal at 45–55 kDa, confirming successful purification and identity as a valid binding receptor (Figure 7).

**Figure 8:**
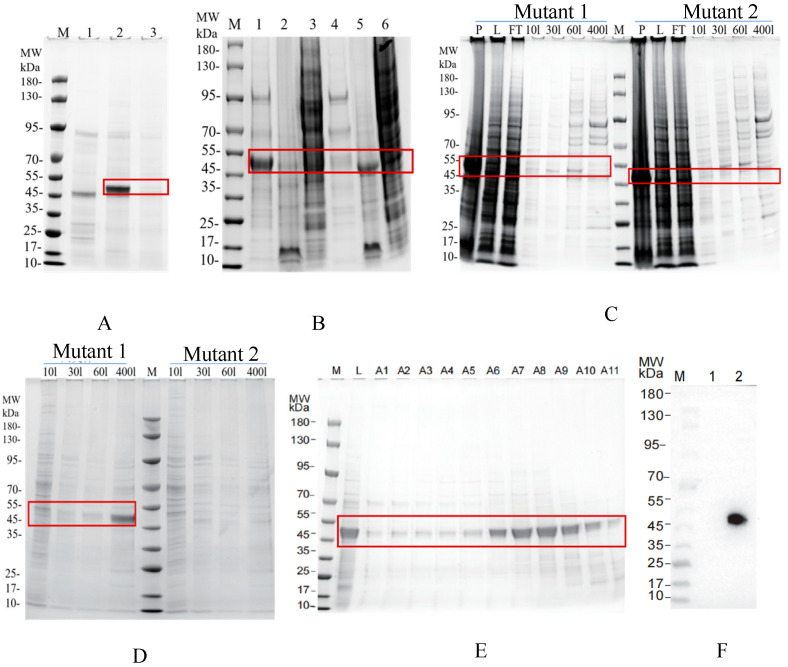
Purification of the mutant 118I. (**A**) Presents the extracellular secreted proteins collected from insect cells. In this panel, M represents the protein marker, 1 is the negative control, 2 corresponds to mutant 118I, and 3 corresponds to mutant 90N93Y. (**B**) Shows the analysis of intracellular proteins. Lanes 1–3 correspond to mutant 118I: 1 represents extracellular protein, 2 is the cell pellet, and 3 is the supernatant of the intracellular fraction. Lanes 4–6 correspond to mutant 90N93Y: 4 represents extracellular protein, 5 is the cell pellet, and 6 is the supernatant of the intracellular fraction. (**C**) Displays the purification results of extracellular secreted proteins. (**D**) Shows the purification results of intracellular proteins. In these panels, M represents the protein marker, P indicates the pellet, L denotes the supernatant, and FT refers to the flow-through fraction. Eluates labeled 10l, 30l, 60l, and 400l correspond to fractions eluted with 10 mM, 30 mM, 60 mM, and 400 mM imidazole, respectively. (**E**) Presents the fractions A1–A11 collected based on elution time or volume during SEC. (**F**) Presents the Western blot analysis, where M represents the protein marker, lane 1 represents the negative control, and 2 corresponds to mutant 118I. Western Blot original pictures see Figures S1–S3.

The 118I mutant of CR1-like CCPs 1-3 was also expressed in insect cells and purified to validate the role of Ile118. SDS-PAGE analysis showed the mutant was soluble in extracellular fractions (45–55 kDa band) and successfully purified via Ni-NTA and SEC; Western blot confirmed the Flag tag and correct molecular weight. In contrast, the 90N93Y mutant formed insoluble intracellular aggregates and could not be purified (Table 1, Figure 8).

**Figure 9:**
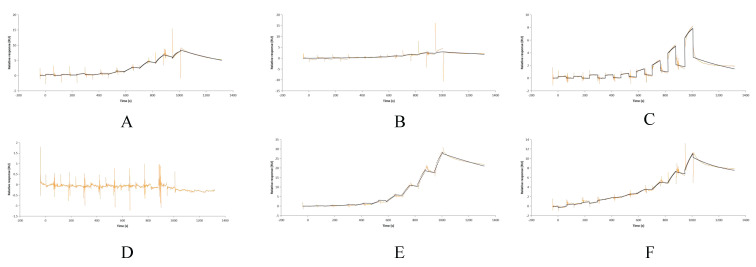
SPR sensing maps of CR1-like and C4b. (**A**) Shows the SPR results for the interaction between CR1-like CCPs 1-3 and C4b α: yellow lines represent the binding response curves of CR1-like CCPs 1-3 (at gradient concentrations 1.56 × 10^−2^–4 μM) with immobilized C4b α; black lines represent the negative control (binding response of bovine serum albumin, BSA, with C4b α, used to exclude non-specific binding). (**B**) Presents the SPR results for the interaction between CR1-like CCPs 1-3 and C4b β: yellow lines = CR1-like CCPs 1-3 (gradient concentrations) binding to C4b β; black lines = BSA binding to C4b β (negative control). (**C**) Displays the SPR results for the interaction between CR1-like CCPs 12-14 and C4b α: yellow lines = CR1-like CCPs 12-14 (gradient concentrations) binding to C4b α; black lines = BSA binding to C4b α (negative control). (**D**) Illustrates the SPR results for the interaction between CR1-like CCPs 12-14 and C4b β: yellow lines = CR1-like CCPs 12-14 (gradient concentrations) binding to C4b β; black lines = BSA binding to C4b β (negative control, no specific binding observed). (**E**) Shows the SPR results for the interaction between mutant 118I and C4b α: yellow lines = mutant 118I (gradient concentrations) binding to C4b α; black lines = BSA binding to C4b α (negative control). (**F**) Presents the SPR results for the interaction between mutant 118I and C4b β: yellow lines = mutant 118I (gradient concentrations) binding to C4b β; black lines = BSA binding to C4b β (negative control).

Surface plasmon resonance (SPR) assays were performed to measure the binding affinity between CR1-like fragments (or the 118I mutant) and C4b chains. Concentration-dependent binding signals were observed for CR1-like CCPs 1-3 with both the C4b α and β chains, while CR1-like CCPs 12-14 showed binding signals only with the C4b α chain. Importantly, the 118I mutant exhibited significantly reduced binding signals to the C4b β chain compared to wild-type CR1-like CCPs 1-3, while its binding to the C4b α chain remained barely unchanged (Figure 9).

**Figure 10:**
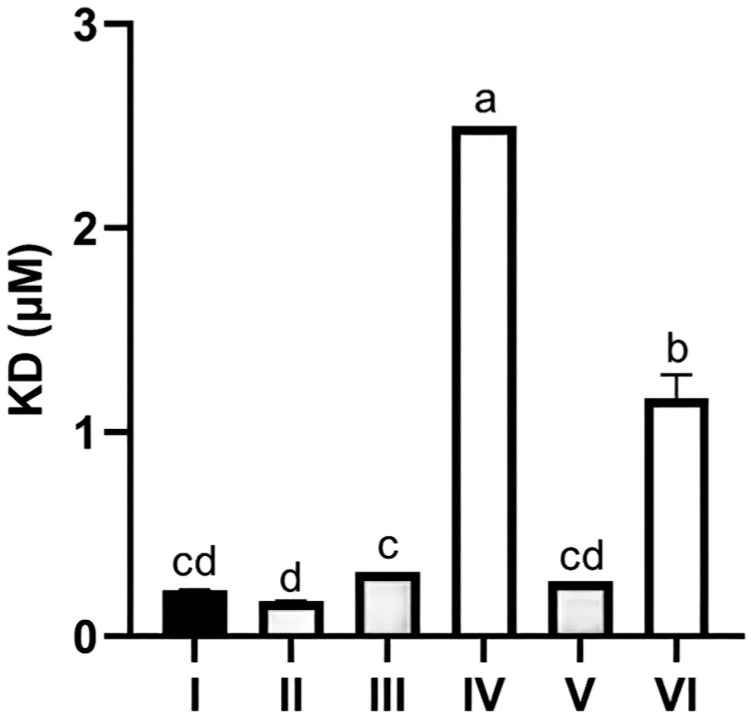
Dissociation constants (KD) of protein interactions between CR1-like CCPs, its mutant, and C4b chains. Groups: I = CR1-like CCPs 1-3 + C4b α; II = CR1-like CCPs 1-3 + C4b β; III = CR1-like CCPs 12-14 + C4b α; IV = CR1-like CCPs 12-14 + C4b β; V = Mutant 118I + C4b α; VI = Mutant 118I + C4b β. Data are mean ± SD from three independent experiments. Different letters (a, b, c, cd, and d) denote significant inter-group differences by one-way ANOVA.

Quantitative analysis of dissociation constants (KD) further confirmed these observations. CR1-like CCPs 1-3 bound C4b α with a KD of 0.226 ± 0.021 μM and C4b β with a KD of 0.173 ± 0.015 μM, indicating high affinity. The 118I mutant showed no significant change in KD for C4b α (0.271 ± 0.019 μM) but a 6.9-fold increase in KD for C4b β (1.2 ± 0.12 μM, *p* < 0.01), confirming that Ile118 is critical for the binding affinity between CR1-like and the C4b β chain (Figure 10).

## 4. Discussion

### 4.1. Functional Mechanisms of CR1-like-C4b Interaction in Complement Regulation and Immune Adhesion

The complement system is a core component of innate immunity, mediating microbial clearance and tissue homeostasis via cytokine/chemokine upregulation. Complement C4, a key molecule in classical/lectin pathways, is cleaved into C4b to initiate opsonization and pathogen clearance. Human CR1 CCPs 1-3 are known to bind C4b [24], and this study explored the functional homology of porcine CR1-like.

We confirmed that PRRSV activates porcine complement C4 in fresh serum, leading to C4b generation—evidenced by reduced C4 activity and C4b-mediated RBC-PRRSV immune adhesion (immunogold-labeled colloidal gold particles observed via electron microscopy). BLAST analysis identified three porcine CR1-like fragments (CCPs 1-3/12-14/19-21) with 58–63% identity to the human CR1 C4b-binding domain, and yeast two-hybrid assays validated their interaction with C4b α/β/γ chains. Due to C4b’s large molecular weight, we expressed it as separate chains, which is a study limitation.

AlphaFold 2-generated 3D models of CR1-like CCPs 1-3 and C4b (validated by SAVES v6.0 and proSA) were used for molecular docking and dynamics simulations. Four tools predicted hot spot residues, but sample exhaustion prevented direct functional validation—we inferred mechanisms via experimental data and the literature.

First, complement activation regulation: SPR showed Ile118 mutation in CR1-like CCPs 1-3 increased KD for the C4b β chain from 0.173 μM to 1.2 μM, with molecular simulations confirming Ile118 forms hydrophobic interactions with C4b β chain’s Leu68/Asn70. This suggests CR1-like competes with CD46 for C4b binding [24,26], restricting C3 convertase (C4bC2a) formation to prevent excessive complement activation and inflammatory damage [33].

Second, immune adhesion efficiency: Electron microscopy and yeast two-hybrid/SPR confirmed C4b mediates RBC-PRRSV adhesion, with CR1-like as the C4b receptor. CR1-like-C4b binding enhances RBC avidity for C4b-opsonized PRRSV, and reduced binding of the Ile118 mutant highlights key residues’ role in adhesion efficiency—analogous to human CR1’s regulation of RBC-mediated pathogen clearance [19,24].

### 4.2. Roles of RBC-PRRSV Adhesion in PRRSV Pathogenesis and Immune Evasion

#### 4.2.1. Facilitation of PRRSV Pathogenesis via RBC-Mediated Viral Dissemination

The activity-dependent CR1-like-C4b adhesion (SPR-verified KD = 0.173 μM for CCPs 1-3/C4b β chain; 6.9-fold KD increase after Ile118 mutation) promotes PRRSV pathogenesis. As the most abundant blood cells, RBCs act as “mobile carriers” for C4b-opsonized PRRSV, efficiently transporting it to target organs (lungs and lymph nodes) via circulation. This enhances PRRSV’s tropism for alveolar macrophages, exacerbating interstitial pneumonia and systemic inflammation—consistent with PRRSV’s respiratory tropism and rapid dissemination. The Ile118 mutation-induced adhesion reduction suggests targeting this axis may impair viral spread, providing a candidate for pathogenicity validation in mutant animal models.

#### 4.2.2. Contribution to PRRSV Immune Evasion via Antigen Masking and Complement Modulation

Beyond known PRRSV escape strategies (interferon inhibition and MHC downregulation), RBC-PRRSV adhesion represents a novel pathway. C4b-opsonized PRRSV binds RBC CR1-like to mask antigen epitopes, evading macrophage/dendritic cell recognition. Additionally, CR1-like’s complement regulatory activity inhibits local complement overactivation, reducing inflammatory cell (neutrophil) recruitment and creating an “immune sanctuary” for viral survival. This aligns with flaviviruses’ NS1-mediated C4 binding and escape [18], but our study is the first to report RBC-mediated complement-dependent evasion.

Collectively, the CR1-like-C4b axis dual-regulates PRRSV pathogenesis and evasion. Targeting this interaction may simultaneously inhibit viral dissemination and escape, offering a promising anti-PRRSV translational direction.

### 4.3. Technical Challenges and Optimization Strategies in Experimental Processes

C4b α/β chains were expressed in *E. coli* and CR1-like CCPs 1-3/118I mutant in insect cells. Tag removal caused protein instability and diffuse bands, so tagged proteins were used—with ExPASy-Prot Param predicting ~40 kDa molecular weight (larger apparent size via SDS-PAGE due to glycosylation). The 90N93Y mutant formed insoluble aggregates and was unobtainable.

Recombinant protein yield/purity variability (common in multi-domain complement proteins [33,40]) stemmed from the following: (1) *E. coli*’s tendency for C4b β chain (≈75 kDa) misfolding/inclusion bodies and insect cell batch-to-batch glycosylation differences [33]; (2) minor induction condition variations (±2 °C, 0.1–0.5 mM IPTG, MOI) causing 15–20% yield fluctuations [40]; (3) partial proteolysis during Ni-NTA purification (faint 10–15 kDa bands), despite protease inhibitors.

This variability only slightly interfered with results: SPR KD values for CCPs 1-3/C4b α/β showed <10% variation (0.226 ± 0.021 μM/0.173 ± 0.015 μM), and degradation products (<5% of total protein) did not bind C4b [35]. Key limitations included C4b’s separate-chain expression (α/β only) and CR1-like’s partial fragment expression (CCPs 1-3/12-14; CCPs 12-14 from our lab’s pre-validated stock [33]). SPR assays only analyzed individual C4b chains (full-length ≈ 204 kDa is difficult to express solubly [33]), with CCPs 1-3 binding C4b α/β (KD = 0.226/0.173 μM), CCPs 12-14 binding only C4b α (KD = 0.317 μM), and Ile118 mutant showing a 6.9-fold higher KD for C4b β (1.2 μM, *p* < 0.01). Future full-length C4b-CR1-like interaction studies could use (1) baculovirus–insect cell co-expression [40]; (2) fragment complementation; and (3) BLI (more suitable for large molecules [35]).

An additional challenge was CR1-like CCPs 1-3/118I instability after tag removal, forcing tagged protein use for SPR—this may have minorly interfered with physiological interaction validation, though core binding trends remained unaltered.

Optimization strategies for future studies: (1) porcine protein-adapted vectors (pET-32a, 6 × His-SUMO) to reduce tag interference [33]; (2) optimized induction (16 °C, 0.1–0.5 mM IPTG, 16–20 h) to minimize inclusion bodies [40]; (3) SEC post Ni-NTA purification + PMSF to reduce aggregation/degradation.

### 4.4. Future Research Directions to Validate In Vivo Functions

To validate functional inferences, CRISPR-Cas9-generated porcine CR1-like mutant cell lines (Ile118Ala, Asn90Ala) or transgenic pigs should assess the following: (1) mutant effects on complement activation markers (C3a and C5a); (2) in vitro/in vivo PRRSV clearance via RBC immune adhesion; (3) lung inflammation and pathological changes in infected mutants. These will clarify CR1-like-C4b’s in vivo role in anti-PRRSV immunity.

Two additional in vitro-derived hypotheses require investigation: (1) full-length C4b (α/β/γ assembled) may have higher affinity for CR1-like than individual chains (CCPs 1-3 bind α/β strongly), requiring soluble full-length C4b expression [4.3]; (2) functional roles of predicted key residues (Tyr136 in CCPs 1-3 and Leu15 in CCPs 12-14) remain unvalidated—Ala-scanning mutagenesis should clarify their contributions.

### 4.5. Translational Implications: Therapeutic Potential Targeting the CR1-like-C4b Axis

Based on CR1-like-C4b interaction data, two anti-PRRSV strategies are proposed: (1) Small-molecule inhibitors: Virtual screening (ZINC database) of compounds blocking CR1-like-C4b binding, using interaction models and key residues (Ile118, Tyr136)—analogous to human CR1-C3b inhibitor design [26]; (2) anti-CR1-like monoclonal antibodies targeting CCPs 1-3/12-14, which may block PRRSV-C4b-RBC adhesion and reduce lung inflammation—specificity is critical to avoid cross-reactivity with other porcine complement receptors [33].

Key challenges and solutions: (1) Poor drug delivery: Lipid nanoparticles enhance inhibitor accumulation in PRRSV-infected lungs [40]; (2) Antibody cross-reactivity: Humanized/camelid single-domain antibodies reduce off-target binding [35]. These strategies require further validation but provide a translational framework for PRRSV control.

## 5. Conclusions

In vitro, fresh serum mediates C4b deposition on PRRSV via complement activation. Porcine erythrocyte CR1-like molecules specifically bind C4b through CCPs 1-3, 12-14, and 19-21, facilitating immune adhesion to PRRSV. Mutation of isoleucine 118 (118I) in CR1-like CCPs 1-3 impairs C4b binding, highlighting its critical role in complement-mediated erythrocyte immune adhesion.

## Figures and Tables

**Table 1:** Yeast two-hybrid (Y2H) interaction assays between porcine CR1-like fragments and C4b chains.

Bait Vector	Bait Fragment	Prey Vector	Prey Fragment	SD/-Leu Growth	SD/-Trp Growth	DDO Growth (SD/-Leu/-Trp)	DDO/X Growth (SD/-Leu/-Trp/X-α-Gal)	DDO/X/A Growth (SD/-Leu/-Trp/X-α-Gal/AbA)	Notes
pGBKT7-53	Positive control insert	pGADT7-T	Positive control insert	+	+	+	+ (Blue)	+ (Blue)	Positive control; validates assay system
pGBKT7-Lam	Negative control insert	pGADT7-T	Positive control insert	−	−	−	−	−	Negative control; excludes non-specific interactions
pGBKT7	Empty vector	pGADT7	Empty vector	+	+	+	+ (Blue)	−	No self-activation; negative control for vectors
pGBKT7-CR1-like	CCPs 1-3	pGADT7-C4b	α chain	+	+	+	+ (Blue)	+ (Blue)	Vigorous growth
pGBKT7-CR1-like	CCPs 1-3	pGADT7-C4b	β chain	+	+	+	+ (Blue)	+ (Blue)	Slightly weaker growth
pGBKT7-CR1-like	CCPs 1-3	pGADT7-C4b	γ chain	+	+	+	+ (Blue)	+ (Blue)	Slightly weaker growth
pGBKT7-CR1-like	CCPs 12-14	pGADT7-C4b	α chain	+	+	+	+ (Blue)	+ (Blue)	Slightly weaker growth
pGBKT7-CR1-like	CCPs 12-14	pGADT7-C4b	β chain	+	+	+	+ (Blue)	+ (Blue)	Vigorous growth
pGBKT7-CR1-like	CCPs 12-14	pGADT7-C4b	γ chain	+	+	+	+ (Blue)	+ (Blue)	Slightly weaker growth
pGBKT7-CR1-like	CCPs 19-21	pGADT7-C4b	α chain	+	+	+	+ (Blue)	+ (Blue)	Slightly weaker growth
pGBKT7-CR1-like	CCPs 19-21	pGADT7-C4b	β chain	+	+	+	+ (Blue)	+ (Blue)	Slightly weaker growth
pGBKT7-CR1-like	CCPs 19-21	pGADT7-C4b	γ chain	+	+	+	+ (Blue)	+ (Blue)	Slightly weaker growth

Notes: Growth notation: “+” = Growth observed; “−” = No growth observed. Color notation: “Blue” = Colony turns blue due to α-Galactosidase activity (indicates reporter gene activation from specific interaction).”Vigorous growth” = Colonies larger and denser compared to other experimental groups; “Slightly weaker growth” = Colonies smaller but viable (consistent with specific interaction). All recombinant plasmids (bait/prey) were pre-validated for non-self-activation and non-toxicity (see original Figure 7, Figure 8 and Figure 9), ensuring observed growth/color is due to specific bait–prey interaction.

## Data Availability

The original contributions presented in this study are included in the article/Supplementary Materials. Further inquiries can be directed to the corresponding author(s).

## Supplementary Materials

The following supporting information can be downloaded at https://www.mdpi.com/article/10.3390/vetsci13010033/s1, Figures S1–S3: Western Blot original pictures.
